# Cytokine-Regulation of Na^+^-K^+^-Cl^−^ Cotransporter 1 and Cystic Fibrosis Transmembrane Conductance Regulator—Potential Role in Pulmonary Inflammation and Edema Formation

**DOI:** 10.3389/fimmu.2017.00393

**Published:** 2017-04-07

**Authors:** Sarah Weidenfeld, Wolfgang M. Kuebler

**Affiliations:** ^1^Keenan Research Centre for Biomedical Science, St. Michael’s Hospital, Toronto, ON, Canada; ^2^Institute of Physiology, Charité-Universitätsmedizin Berlin, Berlin, Germany; ^3^Department of Surgery and Physiology, University of Toronto, Toronto, ON, Canada

**Keywords:** lung inflammation, pulmonary edema, CFTR, NKCC1, cytokines

## Abstract

Pulmonary edema, a major complication of lung injury and inflammation, is defined as accumulation of extravascular fluid in the lungs leading to impaired diffusion of respiratory gases. Lung fluid balance across the alveolar epithelial barrier protects the distal airspace from excess fluid accumulation and is mainly regulated by active sodium transport and Cl^−^ absorption. Increased hydrostatic pressure as seen in cardiogenic edema or increased vascular permeability as present in inflammatory lung diseases such as the acute respiratory distress syndrome (ARDS) causes a reversal of transepithelial fluid transport resulting in the formation of pulmonary edema. The basolateral expressed Na^+^-K^+^-2Cl^−^ cotransporter 1 (NKCC1) and the apical Cl^−^ channel cystic fibrosis transmembrane conductance regulator (CFTR) are considered to be critically involved in the pathogenesis of pulmonary edema and have also been implicated in the inflammatory response in ARDS. Expression and function of both NKCC1 and CFTR can be modulated by released cytokines; however, the relevance of this modulation in the context of ARDS and pulmonary edema is so far unclear. Here, we review the existing literature on the regulation of NKCC1 and CFTR by cytokines, and—based on the known involvement of NKCC1 and CFTR in lung edema and inflammation—speculate on the role of cytokine-dependent NKCC1/CFTR regulation for the pathogenesis and potential treatment of pulmonary inflammation and edema formation.

## Introduction

Pulmonary edema, defined as excessive fluid accumulation in the interstitial and air spaces of the lungs, is a life-threatening condition leading to impaired gas exchange and respiratory failure. Depending on the underlying cause, pulmonary edema is distinguished into two types; hydrostatic and permeability-type edema. The most common form of hydrostatic edema is cardiogenic edema which occurs as major complication of left-sided heart failure and is characterized by increased transcapillary hydrostatic pressure gradients between pulmonary vasculature and interstitial space resulting in interstitial lung edema and flooding of the alveoli with protein-poor fluid. Permeability-type lung edema, also referred to as non-cardiogenic pulmonary edema, is defined by an exudation of protein-rich fluid into the alveoli and develops characteristically in the process of inflammatory lung diseases such as the acute respiratory distress syndrome (ARDS) (1, 2). Two processes are critical for the accumulation of protein-rich fluid in the alveolar space: (1) the disruption of endothelial and epithelial barriers leading to increased vascular permeability and (2) the dysregulated expression or impaired function of ion channels in alveolar epithelial cells limiting fluid removal from the distal airspaces. As such, repair of the epithelial cell barrier and effective clearance of fluid from air spaces are essential prerequisites for resolution of pulmonary edema.

Several transporters (specified in the Section “Fluid Transport of Alveolar Epithelium”), expressed on alveolar type I (ATI) and type II (ATII) cells, are involved in active transport of salt and water through the epithelial barrier leading to alveolar fluid clearance (AFC). AFC is impaired in more than 80% of ARDS patients and associated with increased morbidity and mortality (3). Therefore, manipulation of alveolar fluid transport could represent a suitable therapeutically target. However, molecular mechanism resulting in impaired epithelial fluid transport remains unclear. Inflammatory responses involving upregulation of pro-inflammatory cytokines including interleukin-1β (IL-1β), IL-8, tumor necrosis factor-α (TNF-α), and transforming growth factor-β (TGF-β) and their accumulation in BALF and edema fluid are a critical hallmark of ARDS (4–7). In addition to their role in immune responses, these pro-inflammatory mediators are considered to inhibit alveolar fluid transport by regulation of sodium and chloride transporter (7). However, present understandings of mechanisms by which cytokines regulate ion transport are far from complete, with previous work having focused on the regulation of Na^+^ transport via epithelial Na^+^ channel (ENaC) and the Na^+^/K^+^-ATPase. In this review, we propose that cytokine-dependent regulation of Na^+^-K^+^-2Cl^−^ cotransporter 1 (NKCC1) and Cl^−^ channel cystic fibrosis transmembrane conductance regulator (CFTR) may play a critical role in lung edema formation and pulmonary inflammation. To this end, we first outline the general principles of alveolar fluid transport and the role of inflammatory cytokines in lung edema formation, then focus specifically on the role of NKCC1 and CFTR in pulmonary edema and inflammation, and their regulation by cytokines, and finally conclude by proposing a critical role for cytokine-dependent regulation of NKCC1 and CFTR as a novel concept in the pathogenesis of pulmonary edema.

## Regulation of Active Salt and Water Transport and Edema Formation

### Fluid Transport of Alveolar Epithelium

The alveolar epithelium forms a tight barrier between vasculature and air-filled compartment to control movement of protein and fluid under physiological conditions. It comprises ATI cells, which are responsible for gas exchange across the alveolo-capillary barrier, and ATII cells that fulfill several functions, most notably production and release of surfactant (8).

**Figure 1:**
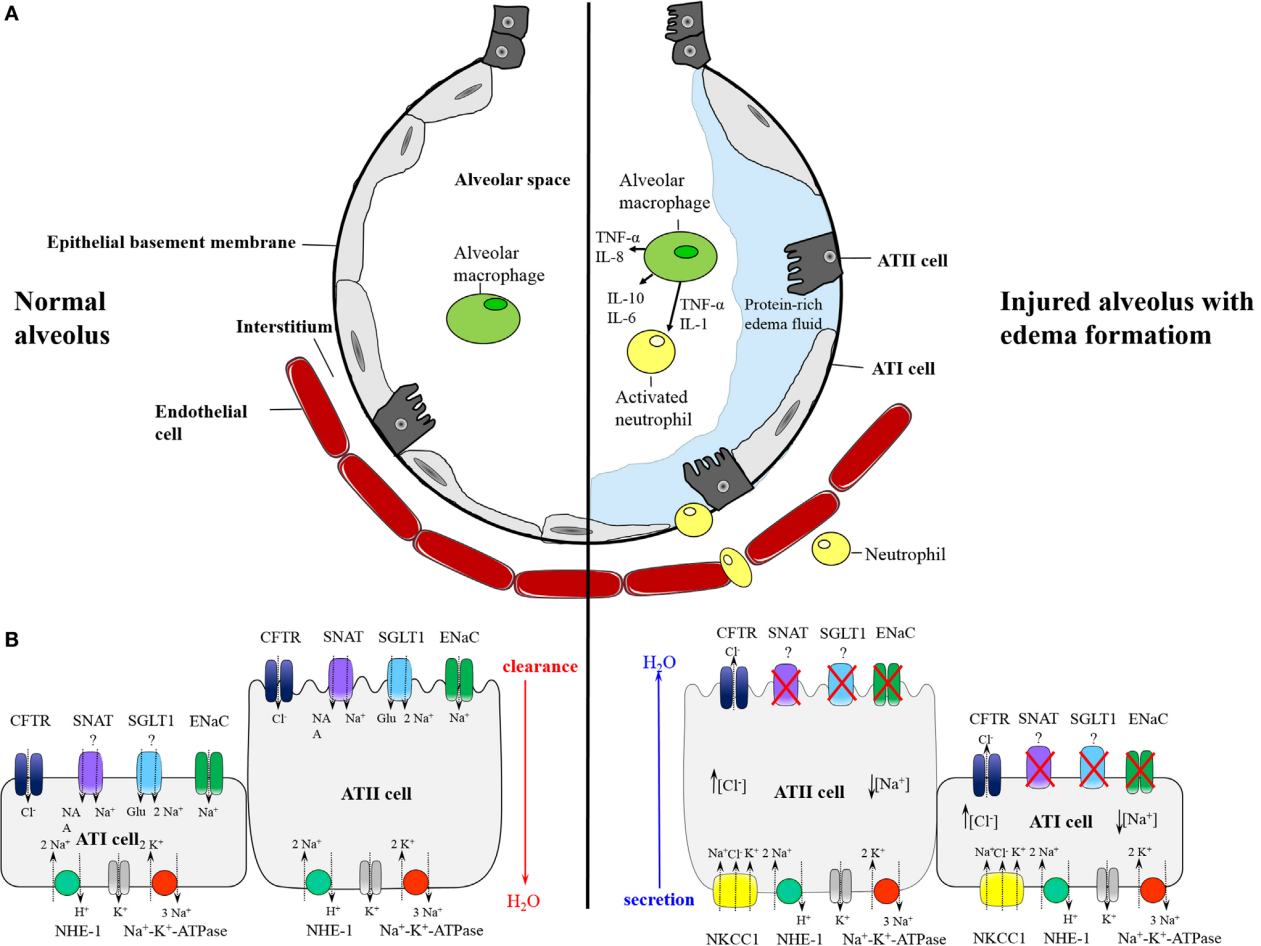
**(A)** Schematic model of the normal alveolus (left) and the injured alveolus (right) with edema formation in inflammatory lung disease. In pulmonary inflammation, the epithelial and endothelial barrier become disrupted leading to influx of protein-rich edema fluid and migration of neutrophils from the vasculature into the alveolar space. In the air space, alveolar macrophages secrete proinflammatory cytokines that stimulate chemotaxis and activate neutrophils which in turn produce and release further cytokines. **(B)** Distribution of epithelial ion transporter and proposed mechanism of alveolar fluid clearance (AFC) (left) and secretion (right). In alveolar type II and presumably also type I cells, AFC is mediated through apical Na^+^ entry by sodium channels like epithelial Na^+^ channel (ENac), sodium-coupled amino acid transporter (SNATs), and sodium glucose transporter (SGLT). Basolateral extrusion is driven by Na^+^-K^+^-ATPase and sodium hydrogen exchanger (NHE). Water and Cl^−^ follow for electroneutrality. In pulmonary edema, ENaC and probably other sodium transporter are inhibited generating a gradient for Na^+^ influx via basolateral NKCC1. Cl^−^ enters in cotransport with Na^+^, and exits along an electrochemical gradient on the apical side through CFTR, resulting in Cl^−^-driven fluid secretion and formation of lung edema.

In the intact lung, AFC constantly moves fluid from the alveolar space across the epithelial barrier into the interstitial space. Na^+^ is actively absorbed on the apical side of alveolar epithelial cells, which is mediated by several sodium channels (Figure 1), most notably the amiloride-sensitive ENaC (9), the sodium glucose transporter (SGLT) (10, 11) and the sodium-coupled neutral amino acid transporter (SNAT) (12–14). On the basolateral surface, Na^+^ extrusion to the interstitial space is driven through the Na^+^-K^+^-ATPase (8) and the Na^+^/H^+^ antiporter (15). For electroneutrality and osmotic balance, Cl^−^ and water follow the electrochemical gradient partly paracellularly and partly through aquaporins (specifically aquaporin 5) (16) and chloride channels, predominantly CFTR (17). Although it was thought that channels and transporter are primarily expressed in ATII cells, recent data demonstrated that ATI cells contain ENaC, CFTR, and the Na^+^-K^+^-ATPase suggesting a role for ATI cells in alveolar fluid transport (9, 18).

In response to lung injury and inflammation, the epithelial barrier becomes disrupted leading to increased influx of protein-rich fluid and formation of pulmonary edema (Figure 1). Furthermore, the physiological protection provided by active salt and water transport is attenuated, resulting in impaired AFC in patients with both cardiogenic (19) and permeability-type (3) lung edema. On top of that, AFC may reverse into active alveolar fluid secretion (AFS), thus promoting rather than resolving edema formation. Recently, work from our group identified basolateral NKCC1 and apical-expressed CFTR as critical for the reversal of an absorptive into a secretory alveolar epithelium by driving Cl^−^ secretion (20). Specifically, we could show that an acute increase in left atrial pressure decreases amiloride-sensitive Na^+^ uptake across the alveolar epithelium and concomitantly stimulates Na^+^ and Cl^−^ uptake via basolateral NKCC1 and Cl^−^ secretion into the alveolar space via apical CFTR, thus effectively reversing Na^+^-driven AFC into Cl^−^ driven AFS. Importantly, inhibition of CFTR and NKCC1 improved AFC and attenuated edema formation. In line with this concept, previous studies have reported similar beneficial effects of NKCC inhibition on edema formation in different organs in that bumetanide reduced cerebral edema formation in response to ischemia (21) and furosemide improved fluid balance and reduced pulmonary edema in ARDS patients (22). Although these effects have traditionally been attributed to diuretic effects of non-specific NKCC inhibitors, improvement of respiratory function by furosemide in lung edema precedes the onset of diuresis (23, 24), suggesting alternative mechanisms such as NKCC/CFTR-mediated AFS in edema formation.

### Pro-Inflammatory Cytokines in Lung Edema Formation

During early stages of injury, the lung is the site of acute inflammatory processes with excessive transepithelial neutrophil migration and continuous release and activation of pro-inflammatory mediators. Pro-inflammatory cytokines, produced by circulating monocytes, alveolar macrophages, and neutrophils, promote recruitment and activation of additional immune cells and inflammatory molecules. A variety of cytokines and growth factors can be detected in BALF and edema fluid of ARDS patients including TNF-α, IL-1β, IL-8, and TGF-β1 (5, 6, 25, 26). These cytokines have been implicated to play a crucial role in the pathophysiology of pulmonary edema formation.

A critical involvement of TNF-α in edema formation has been documented in a series of experimental and clinical studies, which have previously been reviewed in detail (27). In brief, TNF-α reduces the expression of ENaC mRNA in alveolar epithelial cells and thereby decreases amiloride-sensitive sodium uptake (28). However, there is also evidence for a protective effect of TNF-α in pulmonary edema formation, as demonstrated by Borjesson and colleagues who identified in a rat model of intestinal ischemia-reperfusion, a TNF-α-dependent stimulation of AFC in the early phase of injury (29). Similarly, a TNF-dependent and amiloride-sensitive increase in alveolar fluid resorption was detected in a rat model of *Pseudomonas aeruginosa* pneumonia (30).

Inhibition of growth factor TGF-β1 protects wild-type mice from pulmonary edema in a bleomycin-induced lung injury model (31). An increased TGF-β1 activity in distal airways has been shown to promote edema by reducing alveolar epithelial sodium uptake and AFC. This effect of TGF-β1 is considered to be dependent on activation of the MAPK-ERK1/2 pathway resulting in decreased expression of ENaC mRNA (32). A similar effect has been described for IL-1β, which was shown to reduce ENaC expression through p38–MAPK-dependent inhibition of ENaC promoter activity (33). In contrast, an *in vitro* study reported an IL-1β-mediated increase in epithelial repair induced by edema fluid (34).

The chemotactic mediator IL-8 promotes edema formation by blocking AFC (35). Accordingly, inhibition of IL-8 significantly diminishes edema caused by smoke inhalation, acid aspiration, or ischemia-reperfusion injury (36–38).

Overall, there is evidence that cytokines are important regulators of active ion transport and AFC. However, exact regulation of ion channels by inflammatory cytokines may be a complex phenomenon with functional effects depending on temporal and spatial profiles, interdependence between various cytokines, and the presence (*in vivo* situation) or absence (*in vitro* assays) of immune cells. Detailed dissection of these scenarios poses a considerable challenge in terms of both resources and appropriate assays, yet would provide an invaluable platform for a better understanding of the complex crosstalk between inflammation and ion channel activity in a wide range of pulmonary and systemic inflammatory diseases.

## CFTR and NKCC1 in Inflammatory Lung Disease and Pulmonary Edema

### Na^+^-K^+^-Cl^−^ Cotransporter

The Na-K-Cl cotransporter (NKCC) mediates active electroneutral uptake of one Na^+^ and K^+^ with 2 Cl^−^ molecules along an inwardly directed electrochemical gradient for Na^+^ and Cl^−^. Of the two known isoforms, NKCC1 and NKCC2, NKCC1 is found on the basolateral side on epithelial and endothelial cells in several organs, including the alveolar epithelium. In contrast, apically expressed NKCC2 is only present in the kidney epithelium (39). Both isoforms are sensitive to loop diuretics like bumetanide and furosemide, which inhibit ion translocation (40).

To maintain cell shape and integrity during active salt and water secretion, activation of NKCC1 is strictly regulated. Activity of NKCC1 can be induced through hyperosmotic stress (41), low intracellular Na^+^ level, increase in intracellular cAMP, or changes in cell shape, and depends on direct phosphorylation by Ste20-related proline/alanine-rich kinase (SPAK) and oxidative stress responsive kinases (OSR1) (42).

### Cystic Fibrosis Transmembrane Conductance Regulator (CFTR)

CFTR, which has been identified as the mutated gene in cystic fibrosis patients (43), is considered an atypical ATP-binding cassette (ABC) transporter which is activated by phosphorylation and ATP hydrolysis (44). It permits bidirectional transport of Cl^−^ anion depending on the electrochemical gradient. CFTR is expressed on apical membranes of epithelial cells in distal airways and alveolar epithelium, where it mediates Cl^−^ transport to maintain alveolar fluid homeostasis (45). CFTR expression and activation depends on intracellular cAMP or cGMP, which activate PKA and cGKII (46) leading to upregulation of CFTR expression and phosphorylation (47, 48).

### Expression of NKCC1 and CFTR in Inflammatory Lung Diseases

NKCC1 and CFTR are both involved in a variety of biological processes ranging from ion transport to regulation of macrophage activation and modulation of cytokine production (49–52). Of relevance for this review, NKCC1 and CFTR have also been implicated in pulmonary inflammatory processes.

NKCC1 is upregulated in response to Gram-negative bacterial toxins like lipopolysaccharide (LPS) in the lung and kidney (53). Whether this enhanced NKCC1 gene expression is, however, mediated directly by LPS binding to its receptor inducing intracellular signaling or via released inflammatory cytokines like TNF-α after LPS stimulation remains to be elucidated.

Nguyen and colleagues (54) proposed a role for NKCC1 in inflammatory processes in response to *Klebsiella pneumoniae* infection. Mice lacking NKCC1 were protected from bacteremia and lethal sepsis after infection and showed decreased vascular permeability. The number of migrated neutrophils in the air space was increased leading to a reduced number of *K. pneumoniae* in the lung of NKCC1-deficient mice. A potential mechanism that may explain the involvement of NKCC1 in edema formation and neutrophil transmigration was proposed by Matthay and Su (55), who speculated that expression of NKCC1 in endothelial and epithelial cells might be upregulated by inflammatory molecules in response to bacterial infections; however, this hypothesis still awaits functional validation, and regulatory pathways involved remain to be clarified. Along similar lines, a study by Andrade and colleagues reported upregulated NKCC1 expression along with downregulated ENaC in response to *Leptospirosis* infection, a model of sepsis leading to edema formation and ARDS (56). The authors proposed a regulation of transporter expression via JNK and NF-κB pathways during leptospirosis-induced pulmonary edema, yet exact signaling cascades remain unclear.

CFTR is considered an important modulator of inflammatory responses in the lung. Absence of a functional CFTR leads to chronic pulmonary inflammation, as seen in cystic fibrosis patients (57). In a murine model of cerebral and uterine *Chlamydia trachomatis* infection, CFTR mRNA and protein were shown to be upregulated causing increased tissue fluid accumulation and edema formation (58). The authors hypothesized that increased CFTR expression and abnormal fluid accumulation upon *C. trachomatis* infection may depend on increased cytokine release. In pulmonary epithelial cells, CFTR functions as receptor for *P. aeruginosa* internalization leading to CFTR translocation into lipid rafts (59) and NF-κB mediated expression of IL-1β (60). However, the role of CFTR in lung injury and edema formation may be more complex. In a CF mouse model using CFTR-deficient animals, Bruscia and colleagues (61) demonstrated an enhanced pulmonary inflammatory response with elevated cytokine levels in response to chronic LPS exposure. Similarly, Su and coworkers (51) reported aggravated inflammatory cytokine release and edema formation following LPS challenge in mice bearing the human functional CFTR mutation, F508del-CF, or in mice treated with a pharmacological CFTR inhibitor. Importantly, subsequent chimeric experiments in wild-type mice reconstituted with F508del neutrophils or bone marrow, respectively, revealed that pro-inflammatory, pro-edematous effect of functional CF inhibition was attributable to the lack of CFTR on immune cells, specifically neutrophils, rather than epithelial or other parenchymal cells (51). Hence, CFTR may promote both pro-and anti-edematous effects in inflammatory lung disease depending on its site of expression.

Taken together, functional concepts involving NKCC1 and CFTR in fluid transport and edema formation (Figure 1A) in combination with data demonstrating differential regulation of NKCC1 and CFTR in lung injury that coincides with edema formation point toward a critical role for CFTR and NKCC1 in infection-induced pulmonary edema. While molecular mechanisms underlying the regulation of NKCC1 and CFTR by infectious pathogens remain to be elucidated, it is tempting to speculate on a critical role for inflammatory cytokines as putative mediators in the regulation of these channels. As discussed in the following section, multifunctional cytokines like TNF-α, IL-1β, and IL-8 are particularly attractive candidates as key regulators of NKCC1 and CFTR.

### Regulation of NKCC1 and CFTR by Cytokines

Various signaling pathways have been suggested to play a role in NKCC1 activation and expression including WNK, MAP kinase/ERK, p38, and JNK pathways (62–64). Notably, these pathways are also known to be stimulated by cytokines and growth factors like TNF-α and TGF-β, which activate intracellular p38 and JNK pathways (65, 66). In pulmonary inflammation, cytokines may bind to receptors on alveolar epithelial cells and induce intracellular pathways resulting in activation of NKCC1, yet, direct evidence for such an effect in the intact, inflamed lung is presently outstanding. Consistent with this notion, however, NKCC1 mRNA and protein levels were found to be selectively upregulated by TNF-α and IL-1β in endothelial and epithelial cells (53, 67). Conversely, inhibition of TNF-α and IL-1β by hypertonicity was found to be beneficial in cerebral edema, and the functional role of TNF-α and IL-1β in the regulation of NKCC1 expression was validated *in vitro*, leading the authors to propose that TNF-α and IL-1β may directly upregulate NKCC1 expression via JNK- and p38-dependent pathways (67).

For CFTR, the interplay between inflammatory cytokines and channel expression/activity is even more complex. As such, IL-8 has been shown to indirectly regulate activity and biosynthesis of CFTR through inhibition of the β_2_-adrenergic receptor (AR) pathway (35) and subsequent phosphorylation of CFTR via cAMP-mediated PKA activation, which is considered to be essential for AFC (68). Downregulation of β2-AR in ATII cells by IL-8 blocked fluid transport across the alveolar epithelium via inhibition of CFTR phosphorylation and expression (35). Likewise, TGF-β has been proposed to diminish cAMP-driven chloride transport in colonic epithelia via inhibition of CFTR mRNA expression and protein synthesis (69). In 2007, Lee and colleagues (7) investigated the effect of edema fluid on transporter expression in alveolar epithelial cells and showed that cytokine-containing edema fluid decreases expression and activation of CFTR leading to decreased AFC. However, incubation with individual cytokines alone did not alter CFTR expression, suggesting a complex regulatory mechanism.

Other studies, reported an upregulation of CFTR mRNA and protein by IL-1β (70–72). The NF-κB-pathway has been identified to be involved in the IL-1β-dependent increase in CFTR expression (70). In agreement with this effect, CTFR-dependent AFS has been shown to be stimulated by IL-1β and TNF-α in airway submucosal glands via cAMP-dependent activation of PKA (73).

**Figure 2:**
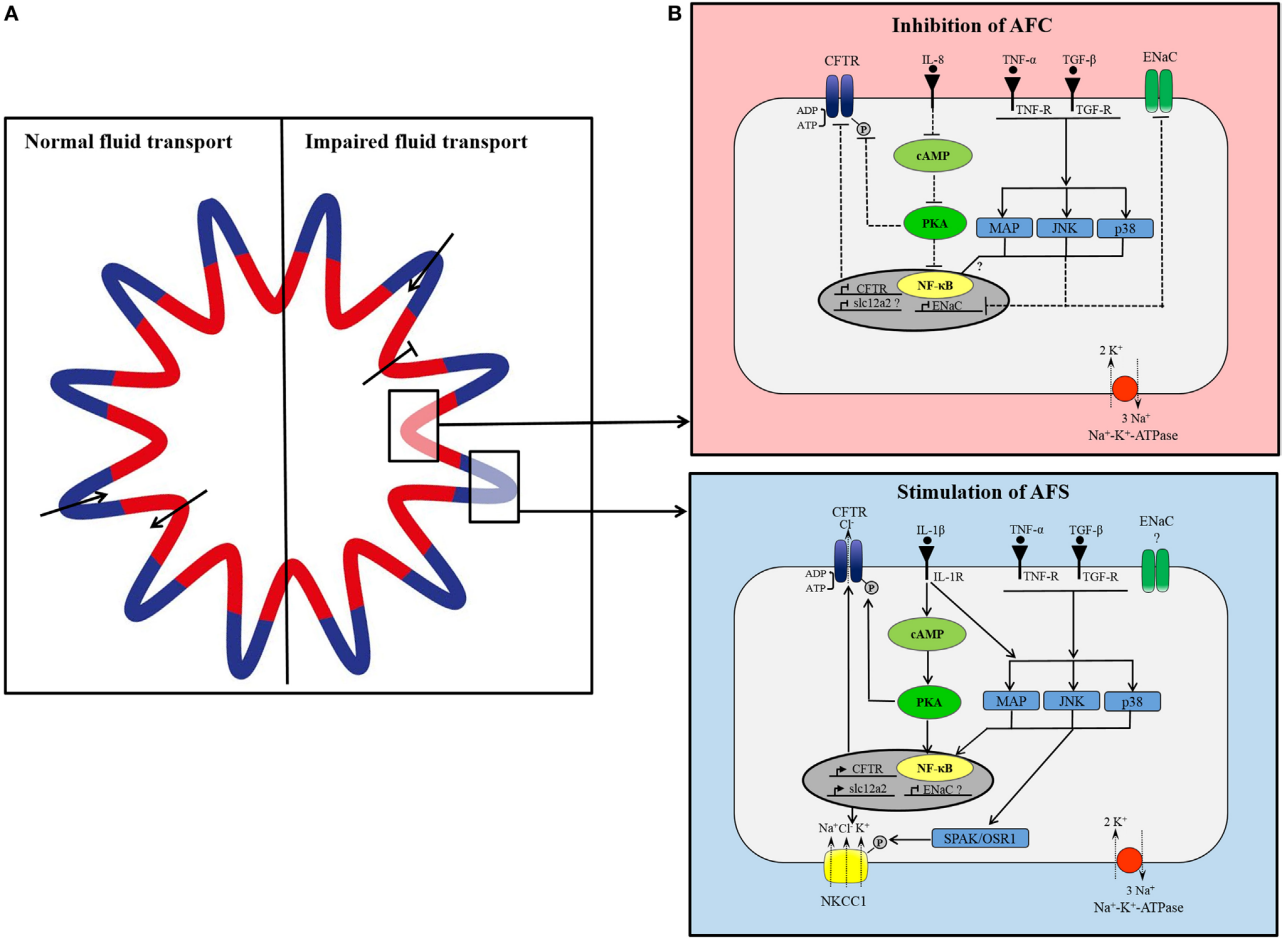
**(A)** Proposed model of organization of small airways. In lung epithelium, independent groups of cells simultaneously secrete and absorb to maintain fluid homeostasis. Under normal conditions, cells located in the pleats secrete fluid (blue) and cells located around the folds concurrently absorb secreted fluid (red). In lung injury, fluid transport in absorptive areas may be blocked while fluid secretion stays intact or increases. **(B)** Simplified, hypothetical concept of differential cytokine-dependent regulation of ion transporter in absorptive (red) and secretory (blue) areas in inflammatory lung disease. In apsorptive areas, AFC is impaired presumably through cytokine-mediated inhibition of CFTR and epithelial Na^+^ channel (ENaC). Proinflammatory cytokines [tumor necrosis factor-α (TNF-α), transforming growth factor-β (TGF-β), interleukin1β (IL-1β)] bind to their receptor inducing intracellular signaling cascades of MAP-kinases, JNK, p38, which prevent expression of ENaC. IL-8 blocks CFTR expression and activation by inhibition of cAMP/PKA pathway. In secretory areas, fluid secretion is stimulated by cytokines. Receptor binding of IL-1β, TNF-α, and TGF-β induces intracellular signaling pathways leading to activation and expression of CFTR and NKCC1.

Contradictions in CFTR regulatory processes and its involvement in fluid transport and edema formation may result from differences in expressed transport system in various cell types, and such heterogeneity may also be present in the pulmonary epithelium. Recently, it has been proposed that the distal airways might be comprised of secreting areas, located in the contra-luminal regions of the pleats, and absorbing areas in the folds (74). Secretion and absorption of fluid is considered to occur simultaneously and independently maintaining the required level of airway surface liquid. Regulation of transporter expression may also vary in these areas. NKCC1 has been found to be abundantly expressed in the pleats of distal airways and less in the folds (75). No change in CFTR expression was detected in different areas (75), which is not surprising assuming CFTR is involved in fluid secretion and absorption. In lung injury, impaired alveolar fluid transport might be triggered by cytokine-mediated differential expression of ion channels including NKCC1 and CFTR that may putatively result in an increased expression and/or activation of NKCC1 and CFTR in secretory epithelia and an inhibition of channels in absorptive areas (Figure 2). While largely speculative at this stage, this hypothetical concept of a spatially (and potentially temporally) differential regulation of ion channels involved in fluid absorption and secretion by inflammatory cytokines highlights the need for expanded research in this fascinating field. An in-depth understanding of changes in ion and fluid flux and their regulation may provide for optimized targets for edema resolution in acute inflammatory lung disease.

## Conclusion

Acute respiratory distress syndrome with edema formation is a serious complication in critically ill patients. Resolution of edema needs strategies to restore epithelial barrier function and improve AFC. Formation of pulmonary edema in inflammatory lung diseases is caused by the loss of endothelial and epithelial barrier and impaired fluid and ion transport across the alveolar epithelium. Pro-inflammatory cytokines like TNF-α, TGF-β1, and IL-1β, which are released and activated in the early phase of lung inflammation, may regulate expression and activity of ion channels involved in fluid transport including NKCC1 and CFTR. Regulation of NKCC1 and CFTR is, however, complex, with discrepant results potentially depending on time profile, cell type, and co-stimulation by different cytokines resulting in a distinct multi-dimensional response that favors AFS while impairing AFC. Manipulation of expression and activity of NKCC1 and CFTR might serve as therapeutic target in inflammatory lung diseases with edema formation.

## Author Contributions

Both authors, SW and WK, meet the following four criteria of authorship: 1. Substantial contributions to the conception or design of the work. 2. Drafting the work and revising it critically for important intellectual content. 3. Final approval of the version to be published; 4. Agreement to be accountable for all aspects of the work in ensuring that questions related to the accuracy or integrity of any part of the work are appropriately investigated and resolved.

## Conflict of Interest Statement

The authors declare that the research was conducted in the absence of any commercial or financial relationships that could be construed as a potential conflict of interest.

## References

[1] Ware LB Matthay MA Acute pulmonary edema. N Engl J Med (2005) 353:2788–96. 10.1056/NEJMcp052699 16382065

[2] Murray JF Pulmonary edema: pathophysiology and diagnosis. Int J Tuberc Lung Dis (2011) 15:155–60, i. 21219673

[3] Ware LB Matthay MA. Alveolar fluid clearance is impaired in the majority of patients with acute lung injury and the acute respiratory distress syndrome. Am J Respir Crit Care Med (2001) 163:1376–83. 10.1164/ajrccm.163.6.2004035 11371404

[4] Ware LB Matthay MA The acute respiratory distress syndrome. N Engl J Med (2000) 342:1334–49. 10.1056/NEJM200005043421806 10793167

[5] Pugin J Verghese G Widmer MC Matthay MA. The alveolar space is the site of intense inflammatory and profibrotic reactions in the early phase of acute respiratory distress syndrome. Crit Care Med (1999) 27:304–12. 10.1097/00003246-199902000-00036 10075054

[6] Olman MA White KE Ware LB Simmons WL Benveniste EN Zhu S Pulmonary edema fluid from patients with early lung injury stimulates fibroblast proliferation through IL-1 induced IL-6 expression. J Immunol (2004) 172:2668–77. 10.4049/jimmunol.172.4.2668 14764742

[7] Lee JW Fang X Dolganov G Fremont RD Bastarache JA Ware LB Acute lung injury edema fluid decreases net fluid transport across human alveolar epithelial type II cells. J Biol Chem (2007) 282:24109–19. 10.1074/jbc.M700821200 17580309 PMC2765119

[8] Matthay MA Folkesson HG Clerici C. Lung epithelial fluid transport and the resolution of pulmonary edema. Physiol Rev (2002) 82:569–600. 10.1152/physrev.00003.2002 12087129

[9] Johnson MD Bao H-F Helms MN Chen X-J Tigue Z Jain L Functional ion channels in pulmonary alveolar type I cells support a role for type I cells in lung ion transport. Proc Natl Acad Sci U S A (2006) 103:4964–9. 10.1073/pnas.0600855103 16549766 PMC1458778

[10] de Prost N Saumon G. Glucose transport in the lung and its role in liquid movement. Respir Physiol Neurobiol (2007) 159:331–7. 10.1016/j.resp.2007.02.014 17369109

[11] Basset G Crone C Saumon G. Fluid absorption by rat lung in situ: pathways for sodium entry in the luminal membrane of alveolar epithelium. J Physiol (1987) 384:325–45. 10.1113/jphysiol.1987.sp016457 3116209 PMC1192265

[12] Clerici C Soler P Saumon G. Sodium-dependent phosphate and alanine transports but sodium-independent hexose transport in type II alveolar epithelial cells in primary culture. Biochim Biophys Acta (1991) 1063:27–35. 10.1016/0005-2736(91)90349-D 2015259

[13] Brown SE Kim KJ Goodman BE Wells JR Crandall ED. Sodium-amino acid cotransport by type II alveolar epithelial cells. J Appl Physiol (1985) 59:1616–22. 4066594 10.1152/jappl.1985.59.5.1616

[14] Michaut P Planes C Escoubet B Clement A Amiel C Clerici C. Rat lung alveolar type II cell line maintains sodium transport characteristics of primary culture. J Cell Physiol (1996) 169:78–86. 10.1002/(SICI)1097-4652(199610)169:1<78:AID-JCP8>3.0.CO;2-B 8841424

[15] Nord EP Brown SE Crandall ED. Characterization of Na^+^-H^+^ antiport in type II alveolar epithelial cells. Am J Physiol (1987) 252:C490–8. 3034070 10.1152/ajpcell.1987.252.5.C490

[16] Dobbs LG Gonzalez R Matthay MA Carter EP Allen L Verkman AS. Highly water-permeable type I alveolar epithelial cells confer high water permeability between the airspace and vasculature in rat lung. Proc Natl Acad Sci U S A (1998) 95:2991–6. 10.1073/pnas.95.6.2991 9501203 PMC19682

[17] Sartori C Matthay MA. Alveolar epithelial fluid transport in acute lung injury: new insights. Eur Respir J (2002) 20:1299–313. 10.1183/09031936.02.00401602 12449188

[18] Ridge KM Olivera WG Saldias F Azzam Z Horowitz S Rutschman DH Alveolar type 1 cells express the alpha2 Na,K-ATPase, which contributes to lung liquid clearance. Circ Res (2003) 92:453–60. 10.1161/01.RES.0000059414.10360.F2 12600893

[19] Verghese GM Ware LB Matthay BA Matthay MA. Alveolar epithelial fluid transport and the resolution of clinically severe hydrostatic pulmonary edema. J Appl Physiol (1999) 87:1301–12. 10517756 10.1152/jappl.1999.87.4.1301

[20] Solymosi EA Kaestle-Gembardt SM Vadász I Wang L Neye N Chupin CJA Chloride transport-driven alveolar fluid secretion is a major contributor to cardiogenic lung edema. Proc Natl Acad Sci U S A (2013) 110:E2308–16. 10.1073/pnas.1216382110 23645634 PMC3690871

[21] O’Donnell ME Tran L Lam TI Liu XB Anderson SE Bumetanide inhibition of the blood-brain barrier Na-K-Cl cotransporter reduces edema formation in the rat middle cerebral artery occlusion model of stroke. J Cereb Blood Flow Metab (2004) 24:1046–56. 10.1097/01.WCB.0000130867.32663.90 15356425

[22] National Heart, Lung, and Blood Institute Acute Respiratory Distress Syndrome (ARDS) Clinical Trials Network Wiedemann HP Clinic C Wheeler AP Bernard GR University V Comparison of two fluid-management strategies in acute lung injury. N Engl J Med (2006) 354:2564–75. 10.1056/NEJMoa062200 16714767

[23] Biddle TL Yu PN. Effect of furosemide on hemodynamics and lung water in acute pulmonary edema secondary to myocardial infarction. Am J Cardiol (1979) 43:86–90. 10.1016/0002-9149(79)90049-3 758775

[24] Ali J Chernicki W Wood LD. Effect of furosemide in canine low-pressure pulmonary edema. J Clin Invest (1979) 64:1494–504. 10.1172/JCI109608 500821 PMC371299

[25] Miller EJ Cohen AB Matthay MA. Increased interleukin-8 concentrations in the pulmonary edema fluid of patients with acute respiratory distress syndrome from sepsis. Crit Care Med (1996) 24:1448–54. 10.1097/00003246-199609000-00004 8797614

[26] Kubo K Hanaoka M Hayano T Miyahara T Hachiya T Hayasaka M Inflammatory cytokines in BAL fluid and pulmonary hemodynamics in high-altitude pulmonary edema. Respir Physiol (1998) 111:301–10. 10.1016/S0034-5687(98)00006-1 9628235

[27] Yang G Hamacher J Gorshkov B White R Sridhar S Verin A The dual role of TNF in pulmonary edema. J Cardiovasc Dis Res (2010) 1:29–36. 10.4103/0975-3583.59983 21188088 PMC3004168

[28] Dagenais A Fréchette R Yamagata Y Yamagata T Carmel J-F Clermont M-E Downregulation of ENaC activity and expression by TNF-α in alveolar epithelial cells. Am J Physiol Lung Cell Mol Physiol (2004) 286:L301–11. 10.1152/ajplung.00326.2002 14514522

[29] Börjesson A Norlin A Wang X Andersson R Folkesson HG. TNF-alpha stimulates alveolar liquid clearance during intestinal ischemia-reperfusion in rats. Am J Physiol Lung Cell Mol Physiol (2000) 278:L3–12. 10645884 10.1152/ajplung.2000.278.1.L3

[30] Rezaiguia S Garat C Delclaux C Meignan M Fleury J Legrand P Acute bacterial pneumonia in rats increases alveolar epithelial fluid clearance by a tumor necrosis factor-alpha–dependent mechanism. J Clin Invest (1997) 99:325–35. 10.1172/JCI119161 9006001 PMC507800

[31] Pittet JF Griffiths MJ Geiser T Kaminski N Dalton SL Huang X TGF-beta is a critical mediator of acute lung injury. J Clin Invest (2001) 107:1537–44. 10.1172/JCI11963 11413161 PMC200192

[32] Frank J Roux J Kawakatsu H Su G Dagenais A Berthiaume Y Transforming growth factor-β1 decreases expression of the epithelial sodium channel αENaC and alveolar epithelial vectorial sodium and fluid transport via an ERK1/2-dependent mechanism. J Biol Chem (2003) 278:43939–50. 10.1074/jbc.M304882200 12930837

[33] Roux J Kawakatsu H Gartland B Pespeni M Sheppard D Matthay MA Interleukin-1β decreases expression of the epithelial sodium channel α-subunit in alveolar epithelial cells via a p38 MAPK-dependent signaling pathway. J Biol Chem (2005) 280:18579–89. 10.1074/jbc.M410561200 15755725

[34] Geiser T Atabai K Jarreau PH Ware LB Pugin J Matthay MA. Pulmonary edema fluid from patients with acute lung injury augments in vitro alveolar epithelial repair by an IL-1beta-dependent mechanism. Am J Respir Crit Care Med (2001) 163:1384–8. 10.1164/ajrccm.163.6.2006131 11371405

[35] Roux J McNicholas CM Carles M Goolaerts A Houseman BT Dickinson DA IL-8 inhibits cAMP-stimulated alveolar epithelial fluid transport via a GRK2/PI3K-dependent mechanism. FASEB J (2013) 27:1095–106. 10.1096/fj.12-219295 23221335 PMC3574281

[36] De Perrot M Sekine Y Fischer S Waddell TK Mcrae K Liu M Interleukin-8 release during early reperfusion predicts graft function in human lung transplantation. Am J Respir Crit Care Med (2002) 165:211–5. 10.1164/rccm2011151 11790657

[37] Laffon M Pittet JF Modelska K Matthay MA Young DM. Interleukin-8 mediates injury from smoke inhalation to both the lung endothelial and the alveolar epithelial barriers in rabbits. Am J Respir Crit Care Med (1999) 160:1443–9. 10.1164/ajrccm.160.5.9901097 10556103

[38] Modelska K Pittet JF Folkesson HG Broaddus VC Matthay MA. Acid-induced lung injury: protective effect of anti-interleukin-8 pretreatment on alveolar epithelial barrier function in rabbits. Am J Respir Crit Care Med (1999) 160:1450–6. 10.1164/ajrccm.160.5.9901096 10556104

[39] Payne JA Xu JC Haas M Lytle CY Ward D Forbush B. Primary structure, functional expression, and chromosomal localization of the bumetanide-sensitive Na-K-Cl cotransporter in human colon. J Biol Chem (1995) 270:17977–85. 10.1074/jbc.270.30.17977 7629105

[40] Russell JM. Sodium-potassium-chloride cotransport. Physiol Rev (2000) 80:211–76. 10617769 10.1152/physrev.2000.80.1.211

[41] Liedtke CM Cole TS. Activation of NKCC1 by hyperosmotic stress in human tracheal epithelial cells involves PKC-delta and ERK. Biochim Biophys Acta (2002) 1589:77–88. 10.1016/S0167-4889(01)00189-6 11909643

[42] Hannemann A Flatman PW. Phosphorylation and transport in the Na-K-2Cl cotransporters, NKCC1 and NKCC2A, compared in HEK-293 cells. PLoS One (2011) 6:e17992. 10.1371/journal.pone.0017992 21464992 PMC3064583

[43] Zeitlin PL Crawfordt I Lut L Woel S Cohen ME Donowitz M CFTR protein expression in primary and cultured epithelia (cystic fibrosis transmembrane conductance regulator/chloride channel). Cell Biol (1992) 89:344–7. 1370353 10.1073/pnas.89.1.344 PMC48233

[44] Riordan JR. Assembly of functional CFTR chloride channels. Annu Rev Physiol (2005) 67:701–18. 10.1146/annurev.physiol.67.032003.154107 15709975

[45] Jiang X Ingbar DH O’Grady SM Adrenergic stimulation of Na^+^ transport across alveolar epithelial cells involves activation of apical Cl channels. Am J Physiol (1998) 275:C1610–20. 9843723 10.1152/ajpcell.1998.275.6.C1610

[46] Hofmann F Ammendola A Schlossmann J. Rising behind NO: cGMP-dependent protein kinases. J Cell Sci (2000) 113:1671–6. 10769198 10.1242/jcs.113.10.1671

[47] Sood R Bear C Auerbach W Reyes E Jensen T Kartner N Regulation of CFTR expression and function during differentiation of intestinal epithelial cells. EMBO J (1992) 11:2487–94. 1378393 10.1002/j.1460-2075.1992.tb05313.x PMC556723

[48] Frizzell RA Hanrahan JW. Physiology of epithelial chloride and fluid secretion. Cold Spring Harb Perspect Med (2012) 2:a009563. 10.1101/cshperspect.a009563 22675668 PMC3367533

[49] Haas M Forbush B. The Na-K-Cl cotransporter of secretory epithelia. Annu Rev Physiol (2000) 62:515–34. 10.1146/annurev.physiol.62.1.515 10845101

[50] Gao Z Su X. CFTR regulates acute inflammatory responses in macrophages. QJM (2015) 108:951–8. 10.1093/qjmed/hcv067 25778108

[51] Su X Looney MR Su H Lee JW Song Y Matthay MA. Role of CFTR expressed by neutrophils in modulating acute lung inflammation and injury in mice. Inflamm Res (2011) 60:619–32. 10.1007/s00011-011-0313-x 21301926 PMC3116128

[52] Rubin BK CFTR is a modulator of airway inflammation. Am J Physiol Lung Cell Mol Physiol (2007) 292:L381–2. 10.1152/ajplung.00375.2006 17012368

[53] Topper JN Wasserman SM Anderson KR Cai J Falb D Gimbrone MA. Expression of the bumetanide-sensitive Na-K-Cl cotransporter BSC2 is differentially regulated by fluid mechanical and inflammatory cytokine stimuli in vascular endothelium. J Clin Invest (1997) 99:2941–9. 10.1172/JCI119489 9185518 PMC508146

[54] Nguyen M Pace AJ Koller BH. Mice lacking NKCC1 are protected from development of bacteremia and hypothermic sepsis secondary to bacterial pneumonia. J Exp Med (2007) 204:1383–93. 10.1084/jem.20061205 17517966 PMC2118609

[55] Matthay MA Su X Pulmonary barriers to pneumonia and sepsis. Nat Med (2007) 13:780–1. 10.1038/nm0707-780 17618264

[56] Andrade L Rodrigues AC Jr Sanches TR Souza RB Seguro AC. Leptospirosis leads to dysregulation of sodium transporters in the kidney and lung. Am J Physiol Renal Physiol (2007) 292:F586–92. 10.1152/ajprenal.00102.2006 16940563

[57] Terheggen-Lagro SW Rijkers GT van der Ent CK. The role of airway epithelium and blood neutrophils in the inflammatory response in cystic fibrosis. J Cyst Fibros (2005) 4:15–23. 10.1016/j.jcf.2005.05.007 15967736

[58] Ajonuma LC He Q Sheung Chan PK Yu Ng EH Fok KL Yan Wong CH Involvement of cystic fibrosis transmembrane conductance regulator in infection-induced edema. Cell Biol Int (2008) 32:801–6. 10.1016/j.cellbi.2008.03.010 18462959

[59] Kowalski MP Pier GB. Localization of cystic fibrosis transmembrane conductance regulator to lipid rafts of epithelial cells is required for *Pseudomonas aeruginosa*-induced cellular activation. J Immunol (2004) 172:418–25. 10.4049/jimmunol.172.1.418 14688350

[60] Reiniger N Lee MM Coleman FT Ray C Golan DE Pier GB. Resistance to *Pseudomonas aeruginosa* chronic lung infection requires cystic fibrosis transmembrane conductance regulator-modulated interleukin-1 (IL-1) release and signaling through the IL-1 receptor. Infect Immun (2007) 75:1598–608. 10.1128/IAI.01980-06 17283089 PMC1865697

[61] Bruscia E Zhang PX Barone C Scholte B Homer RJ Krause D Increased susceptibility of cftr^−/−^ mice to LPS-induced lung remodeling. Am J Physiol Lung Cell Mol Physiol (2016) 310(8):L711–9. 10.1152/ajplung.00284.2015 26851259 PMC4836110

[62] Bachmann O Wüchner K Rossmann H Leipziger J Osikowska B Colledge WH Expression and regulation of the Na^+^-K^+^-2Cl cotransporter NKCC1 in the normal and CFTR-deficient murine colon. J Physiol (2003) 549:525–36. 10.1113/jphysiol.2002.030205 12692180 PMC2342946

[63] Hoorn EJ Nelson JH McCormick JA Ellison DH. The WNK kinase network regulating sodium, potassium, and blood pressure. J Am Soc Nephrol (2011) 22:605–14. 10.1681/ASN.2010080827 21436285 PMC4496838

[64] Jaggi AS Kaur A Bali A Singh N. Expanding spectrum of sodium potassium chloride co-transporters in the pathophysiology of diseases. Curr Neuropharmacol (2015) 13:369–88. 10.2174/1570159X13666150205130359 26411965 PMC4812803

[65] Clark AR Dean JL Saklatvala J The p38 MAPK pathway mediates both antiinflammatory and proinflammatory processes: comment on the article by Damjanov and the editorial by Genovese. Arthritis Rheum (2009) 60:3513–4. 10.1002/art.24919 19877029

[66] Johnson GL Lapadat R Johnson GL Lapadat R Lapadat R Mitogen-activated protein kinase pathways mediated by ERK, JNK, and p38 protein kinases. Science (2002) 298:1911–2. 10.1126/science.1072682 12471242

[67] Huang LQ Zhu GF Deng YY Jiang WQ Fang M Chen CB Hypertonic saline alleviates cerebral edema by inhibiting microglia-derived TNF-α and IL-1β-induced Na-K-Cl cotransporter up-regulation. J Neuroinflammation (2014) 11:102. 10.1186/1742-2094-11-102 24916922 PMC4080704

[68] O’Grady SM Lee SY. Chloride and potassium channel function in alveolar epithelial cells. Am J Physiol Lung Cell Mol Physiol (2003) 284:L689–700. 10.1152/ajplung.00256.2002 12676759

[69] Howe KL Wang A Hunter MM Stanton BA McKay DM TGF-beta down-regulation of the CFTR: a means to limit epithelial chloride secretion. Exp Cell Res (2004) 298:473–84. 10.1016/j.yexcr.2004.04.026 15265695

[70] Brouillard F Bouthier M Leclerc T Clement A Baudouin-Legros M Edelman A NF-kB mediates up-regulation of CFTR gene expression in Calu-3 cells by interleukin-1beta. J Biol Chem (2001) 276:9486–91. 10.1074/jbc.M006636200 11114294

[71] Cafferata EG González-Guerrico AM Giordano L Pivetta OH Santa-Coloma TA. Interleukin-1 beta regulates CFTR expression in human intestinal T84 cells. Biochim Biophys Acta (2000) 1500:241–8. 10.1016/S0925-4439(99)00105-2 10657593

[72] Cafferata EG Guerrico AM Pivetta OH Santa-Coloma TA. NF-kappaB activation is involved in regulation of cystic fibrosis transmembrane conductance regulator (CFTR) by interleukin-1beta. J Biol Chem (2001) 276:15441–4. 10.1074/jbc.M010061200 11278608

[73] Baniak N Luan X Grunow A Machen TE Ianowski JP. The cytokines interleukin-1β and tumor necrosis factor-α stimulate CFTR-mediated fluid secretion by swine airway submucosal glands. Am J Physiol Lung Cell Mol Physiol (2012) 303:L327–33. 10.1152/ajplung.00058.2012 22683572

[74] Shamsuddin AKM Quinton PM Surface fluid absorption and secretion in small airways. J Physiol (2012) 5901515:3561–74. 10.1113/jphysiol.2012.230714 PMC3547270 22547637

[75] Flores-Delgado G Lytle C Quinton PM. Site of fluid secretion in small airways. Am J Respir Cell Mol Biol (2016) 54:312–8. 10.1165/rcmb.2015-0238RC 26562629

